#  Clinical performance of a syndromic panel for direct identification of pathogens and antimicrobial resistance markers in pediatric osteoarticular and pleural space infections

**DOI:** 10.1128/jcm.00621-25

**Published:** 2025-09-02

**Authors:** B. C. Sanchez, H. Sayeed, D. T. Niles, J. J. Dunn

**Affiliations:** 1 Department of Pathology & Immunology, Baylor College of Medicine 189532, Houston, Texas, USA; 2 Department of Pathology and Genomic Medicine, Houston Methodist Research Institute and Houston Methodist Hospital 167626 , Houston, Texas, USA; 3 Department of Pathology and Laboratory Medicine, Weill Cornell Medical College 12295 , New York, New York, USA; 4 Department of Pathology, Texas Children’s Hospital, Houston, Texas, USA; 5 Department of Pediatrics, Baylor College of Medicine 506057, Houston, Texas, USA; Cleveland Clinic, Cleveland, Ohio, USA

**Keywords:** pediatrics, joint infection, empyema, pleural fluid, septic arthritis, multiplex PCR

## Abstract

The recovery of microbial pathogens from sterile body fluids in children poses challenges, including the low sensitivity of conventional culture. Pre-treatment with empiric antimicrobials can render the pathogen non-viable. In such cases, and with fastidious organisms like *Kingella kingae*, molecular methods are useful for identification of the causative agent. This study evaluated the clinical performance of the bioMérieux BIOFIRE Joint Infection (JI) Panel to standard-of-care (SOC) diagnostic techniques in pediatric osteoarticular and pleural fluid and abscess specimens. A total of 136 specimens (77 joint and 59 pleural), were tested with the JI Panel. SOC methods included a lab-developed, real-time PCR assay (Laboratory-developed PCR [LDT-PCR]), routine culture and ancillary testing. Compared to the composite SOC methods, the JI Panel had a positive percent agreement (PPA) of 90.1% and negative percent agreement (NPA) of 99.9% for the detection of on-panel organisms in osteoarticular specimens. For pleural specimens, the JI Panel had a PPA of 93.8% and NPA of 99.6%. False negatives by the JI Panel in both specimen types were detected by the LDT-PCR with cycle thresholds ≥35, which may suggest a low burden of microorganisms in these specimens. The JI Panel demonstrated good performance for the detection of *K. kingae* (PPA = 93.8%) in osteoarticular specimens and *Streptococcus pneumoniae* (PPA = 95.2%) in pleural specimens, the most common pediatric pathogens identified in these respective specimen types. The JI Panel has the potential to impact the treatment and management of pediatric patients, especially in culture-negative cases.

**IMPORTANCE:**

The identification of the causative agents of osteoarticular and pleural space infections in children is challenging, as routine bacterial cultures often yield no growth. The BIOFIRE Joint Infection Panel is a rapid, multiplex PCR assay that detects a wide range of microorganisms and antimicrobial resistance genes. This study demonstrates that the panel has good agreement with standard of care methods for the detection of common pediatric pathogens and may aid in the rapid diagnosis of osteoarticular and pleural space infections in children. However, it is recommended that the assay be used in conjunction with standard bacterial cultures and results interpreted within the context of the clinical presentation, since false-negative and false-positive results may occur.

## INTRODUCTION

Septic arthritis is considered an orthopedic emergency because of its potential for rapid joint destruction with irreversible loss of function, with mortality rates as high as 7%–15% (1, 2). Although all ages can be affected, native joint septic arthritis most commonly occurs in the elderly and very young children. In children, upper respiratory tract infections frequently precede the development of septic arthritis caused by *Haemophilus influenzae* type B and *Kingella kingae,* and oral ulcers are often noted prior to *Kingella* infections. Similar to mild traumatic injuries, these infections are thought to increase the likelihood of progression to bacteremia and seeding of the affected joint (3).

Like septic arthritis, infections of the pleural space are more commonly observed in young children. The most common cause of exudative pleural space infectious processes in children is community-acquired pneumonia, with empyema developing in 2%–12% of children with pneumonia and up to 28% of those requiring hospitalization (4). *Streptococcus pneumoniae* is the most common etiology of bacterial empyema (5), although it is infrequently isolated in culture in infections involving the pleural space (6, 7).

Rapid and accurate diagnosis of infections in sterile body fluids such as synovial (joint) and pleural fluids is essential for the early selection of optimal antibiotic treatment and significantly impacts patient outcomes. However, the recovery of microbial pathogens from sterile body fluids in children using conventional culture techniques poses several challenges, including sensitivities ranging from <25% to 60% (8–10). There may be limited specimen volume and low microbial burden in these specimens. Additionally, prior antimicrobial treatment can render the pathogen non-viable in culture in some cases. In such situations, and particularly with fastidious organisms like *K. kingae*, molecular methods are useful for identification of the causative agent. The bioMérieux BIOFIRE Joint Infection (JI) Panel is a multiplex PCR-based panel that simultaneously detects and identifies 31 organisms and 8 antimicrobial resistance genes (Table S1). The JI Panel is cleared by the U.S. Food and Drug Administration (FDA) for testing of synovial fluid. In this study, we evaluated the clinical performance of the JI Panel to standard-of-care (SOC) diagnostic techniques applied to synovial fluids, and the off-label use of this panel for pathogen identification in pleural fluids, and joint and pleural abscess specimens from pediatric patients.

## MATERIALS AND METHODS

### Clinical specimens

A total of 136 specimens (77 joint and 59 pleural) from pediatric patients archived at −80°C were included in this analysis. Joint fluids and abscesses, bone abscesses, and pleural fluids and abscesses, including two specimens collected using ESwab transport media (Tables S2 and S3), were evaluated for inclusion in the study. Specimens with sufficient volume (>200 µL) to be tested using the bioMérieux BIOFIRE JI Panel and with SOC testing results available in the medical record were included in the study. All SOC testing was performed before specimens were archived at −80°C. SOC testing consisted of aerobic bacterial culture of specimens inoculated on solid media and into aerobic blood culture bottles and interrogation of specimens using a lab-developed, real-time PCR for five bacterial targets (details below). Results of ancillary microbiology testing performed within ±7 days of collection of the osteoarticular and pleural specimens were evaluated if available. Ancillary testing of the same specimen included anaerobic culture, species-specific PCR, and 16S rRNA PCR and sequencing performed at the University of Washington (11, 12), as well as blood cultures.

### Laboratory-developed PCR (LDT-PCR) assay for sterile body fluids

The LDT-PCR assay included primers and probes specific for *Staphylococcus aureus*, methicillin resistance (*mec*A), *K. kingae*, *S. pneumoniae*, *S. pyogenes,* and *Neisseria meningitidis*. The assay was performed on all specimens as described previously (6). Briefly, nucleic acid was extracted using 200 µL of specimen fluid and the Qiagen EZ1 DNA Tissue Kit and the Bacterial Card on the Qiagen EZ1 Advanced XL instrument (Qiagen, Germantown, MD). Multiplex real-time PCR amplification was performed using two master mix reactions, one for detection of *S. aureus* (*Sa442* gene), *S. pneumoniae* (*lytA* gene), and *K. kingae* (*rtxA* gene) and the other for detection of *S. pyogenes* (*speB* gene), *N. meningitidis* (*ctrA* gene), and the *mecA* gene. Amplification and detection were performed on the ABI 7500 real-time PCR system (Applied Biosystems, Waltham, MA).

### Routine bacterial culture

Specimens for aerobic culture were inoculated onto 5% sheep blood, chocolate, and MacConkey agar plates (Remel, Lenexa, KS), and incubated aerobically at 37°C with 5% CO_2_ for up to 5 days. An aliquot of the fluid was also inoculated into a BacT/Alert Virtuo FA Plus blood culture bottle (bioMerieux, Durham, NC) which was incubated on the automated BACT/ALERT VIRTUO blood culture system (bioMerieux) for up to 5 days. Specimens for anaerobic culture were inoculated onto brucella blood agar with 5% sheep blood, Bacteroides bile esculin agar, laked brucella blood agar with kanamycin and vancomycin , and columbia agar with colistin and nalidixic acid (Remel, Lenexa, KS), and incubated anaerobically at 37°C for up to 5 days. Identification of isolated bacterial colonies was performed using the VITEK-MS Matrix Assisted Laser Desorption Ionization Time-of-Flight (MALDI-TOF) instrument (bioMerieux) and biochemical tests as needed according to standard microbiological practices. Antimicrobial susceptibility testing of aerobic organisms was performed using the VITEK2 instrument (bioMerieux).

### Joint Infection (JI) Panel testing

Specimens archived at −80°C were thawed, vortexed, and tested with the JI Panel according to the manufacturer’s instructions. Briefly, 200 µL of specimen was mixed with the sample buffer and injected into the JI Panel pouch for nucleic acid extraction, amplification, and detection. Pouches were then analyzed on the BioFire FilmArray 2.0 or Torch instruments (bioMérieux).

### Data analysis

The results from the JI Panel were compared with those from SOC testing (LDT-PCR and routine aerobic culture) and ancillary tests. Results for each analyte included in the JI Panel were classified as true positive or true negative only when it agreed with SOC and ancillary testing composite results (SOC composite). If more than one organism was detected by the JI Panel in the same specimen, each positive result was compared to SOC composite results and accounted for in the data analysis. The results for anaerobic organisms detected by the JI Panel were only included in the analysis if an anaerobic culture was available for that specimen. Two-by-two data tables were used to determine target-level agreement between the JI Panel and SOC composite results. Analysis of antimicrobial resistance targets was performed by comparing the JI Panel to results of the *mecA* gene included in the LDT-PCR assay, and phenotypic susceptibility results for *S. aureus* isolated in aerobic culture. A true positive for CTX-M was determined if the organism demonstrated phenotypic resistance to third-generation cephalosporins. PPA and NPA along with 95% CIs were calculated using GraphPad Prism version 9.0 for Windows, GraphPad Software, Boston, Massachusetts USA, https://www.graphpad.com/.

## RESULTS

A total of 77 osteoarticular specimens archived at −80°C were thawed and retrospectively tested with the JI Panel. These included 69 synovial fluids, 5 joint abscess, and 3 bone abscess specimens (Table S2). Synovial fluids were primarily collected from knee (*N* = 36), hip (*N *= 12) and ankle (*N *= 8) joints. Of the 77 osteoarticular specimens included in the study, 21 were negative and 56 were positive for organism identification by SOC methods. The JI Panel was positive and agreed with SOC testing results in 50 out of 56 joint specimens amounting to 64 targets detected by the JI Panel out of 71 total possible for an overall PPA of 90.1%. PPA for individual targets ranged from 75% to 100% (Table 1). False negatives by the JI Panel were detected in the LDT-PCR with cycle thresholds (Cts) ≥36.4 and included one *K. kingae* (Ct = 37.5), one methicillin-susceptible *S. aureus* (MSSA) (Ct = 37.4), one methicillin-resistant *S. aureus* (MRSA) (Ct =3 7.6), and one *S*. *pyogenes* (Ct = 36.4). One specimen in which *S. anginosus* was recovered in broth culture only and one specimen positive for *N. gonorrhoeae* by 16S rRNA PCR and sequencing were also negative by the JI Panel. The JI Panel had an overall NPA of 99.9% for osteoarticular specimens, with NPAs for individual targets ranging from 98.7% to 100%. One specimen was positive for *Peptoniphilus* spp. and *Escherichia coli* by the JI Panel, but these organisms were not identified by SOC testing.

**TABLE 1:** Comparison of the BIOFIRE JI Panel and SOC diagnostic techniques in osteoarticular specimens^*b*^

Analyte	PPA			NPA		
	TP/(TP + FN)	%	95% CI	TN/(TN + FP)	%	95% CI
Gram-positive bacteria						
*S. aureus*	12/14	85.7	60–97.5	63/63	100	94.3–100
*Streptococcus* spp.	14/16	87.5	63.9–97.8	61/61	100	94.1–100
*S. pyogenes*	10/11	90.9	62.3–99.5	66/66	100	94.5–100
*S. pneumoniae*	2/2	100	17.8–100	75/75	100	95.2–100
*S. agalactiae*	2/2	100	17.8–100	75/75	100	95.2–100
*Peptoniphilus*	0/0	–^*c*^	–	76/77	98.7	93–99.9
Gram-negative bacteria						
*E. coli*	2/2	100	17.8–100	74/75	98.7	92.8–99.9
*K. kingae*	15/16	93.8	71.7–99.7	61/61	100	94–100
*N. gonorrhoeae*	3/4	75	30–98.7	73/73	100	95–100
*Pseudomonas aeruginosa*	1/1	100	5.1–100	76/76	100	95.2–100
*Salmonella* spp.	2/2	100	17.8–100	75/75	100	95.1–100
*Serratia marcescens*	1/1	100	5.1–100	76/76	100	95.2–100
Other aerobic bacteria and yeast (12)^*a*^	0/0	–	–	924/924	100	99.6–100
Overall	64/71	90.1	81–95.1	1,775/1,777	99.9	99.6–100
AMR genes						
*mecA*	3/4	75	30–98.7	9/9	100	70.1–100
*ctx-m*	1/1	100	5.1–100	5/5	100	56.6–100

^*a*^ Other aerobic bacteria and yeast includes *Enterococcus faecalis*, *Enterococcus faecium*, *S. lugdunensis*, *Citrobacter* spp., *Enterobacter cloacae complex*, *Haemophilus influenzae*, *Klebsiella aerogenes*, *Klebsiella pneumoniae* group, *Morganella morganii*, *Proteus* spp., *Candida* spp., and *Candida albicans*.

^*b*^ PPA: positive percent agreement, NPA: negative percent agreement, TP: true positive, FN: false negative, TN: true negative, FP: false positive, CI: confidence interval, AMR: Antimicrobial resistance.

^*c*^ “–”, no data available.

A total of 59 pleural specimens were retrospectively tested with the JI Panel, including 58 pleural fluids and 1 pleural abscess. Of this total, 25 were negative and 34 were positive for organism identification by SOC methods (Table S3). For pleural specimens, the JI Panel had an overall PPA of 93.8% (60/64) for the targets evaluated in this study. PPAs for individual targets ranged from 50% to 100% (Table 2). MSSA, MRSA, and *S. pneumoniae* were detected in three different samples only by LDT-PCR with Cts ≥35.6, which suggests that a low burden of microorganisms was present in these samples. The overall NPA for the JI Panel with pleural specimens was 99.6%, with NPAs for individual targets ranging from 96.5% to 100% (Table 2). More than one target was identified in four specimens by the JI Panel, three of which included false positive co-detections. A total of five false positives were identified with the JI Panel in four specimens: one *Parvimonas micra*, two *H. influenzae*, one *S. pyogenes,* and one *Streptococcus* spp.

**TABLE 2:** Comparison of the BIOFIRE JI Panel and SOC diagnostic techniques in pleural specimens^*b*^

Analyte	PPA			NPA		
	TP/(TP + FN)	%	95% CI	TN/(TN + FP)	%	95% CI
Gram-positive bacteria						
*S. aureus*	2/4	50	8.9–91.1	55/55	100	93.5–100
*Streptococcus* spp.	29/30	96.7	83.3–99.8	28/29	96.6	82.8–99.8
*S. pyogenes*	6/6	100	60.1–100	52/53	98.1	90–99.9
*S. pneumoniae*	20/21	95.2	78.2–99.8	38/38	100	90.8–100
*P. micra*	1/1	100	5.1–100	57/58	98.3	90.9–99.9
Gram-negative bacteria						
*H. influenzae*	2/2	100	17.8–100	55/57	96.5	88.1–99.4
Other aerobic bacteria and yeast (18)^*a*^	0/0	–^*c*^	–	1,062/1,062	100	99.6–100
Overall	60/64	93.8	85–97.5	1,347/1,352	99.6	99.1–99.8
AMR genes						
*mecA*	1/1	100	5.1–100	1/1	100	5.1–100

^*a*^ Other aerobic bacteria and yeast includes *Enterococcus faecalis, Enterococcus faecium, S. lugdunensis, S. agalactiae*, *Citrobacter *spp.,* Enterobacter cloacae complex*, *Escherichia coli*, *K. kingae, Klebsiella aerogenes, Klebsiella pneumoniae* group, Morganella morganii, *N. gonorrhoeae, Proteus* spp., *Pseudomonas aeruginosa, Salmonella *spp., *Serratia marcescens, Candida *spp., and *Candida albicans*.

^*b*^ PPA: positive percent agreement, NPA: negative percent agreement, TP: true positive, FN: false negative, TN: true negative, FP: false positive, CI: confidence interval, AMR: Antimicrobial resistance.

^*c*^ “–”, no data available.

Overall, the JI Panel demonstrated a target-level PPA of 91.9%, an NPA of 99.8%, and a concordance rate of 99.4% with SOC composite results across both types of samples. Off-panel organisms, including potential bacterial pathogens and members of normal skin and respiratory flora, were isolated by culture but not detected by the JI Panel in eight specimens (Table 3).

**TABLE 3:** Off-panel organisms identified by SOC diagnostic techniques (*N* = 8 specimens^*a*^)

Organism or group	Osteoarticular	Pleural
*Acinetobacter* spp.	1	0
*Bacillus* spp.	2	0
*S. epidermidis*	0	1
*S. auricularis*	1	0
*Micrococcus luteus*	1	0
*Prevotella* spp.	1	2
*Granulicatella adiacens*	0	1
*Actinomyces odolontolyticus*	0	1

^*a*^ More than one organism was isolated from three different specimens.

## DISCUSSION

For osteoarticular specimens the JI Panel demonstrated an acceptable overall PPA of 90.1% and NPA of 99.9% for the detection of on-panel target organisms from our pediatric population. These findings align with large prospective multicenter studies analyzing primarily synovial fluids from adults, which reported PPAs ranging from 90.5% to 92% and NPAs ranging from 84.7% to 98.5% (13, 14). An important advantage of utilizing molecular methods for the diagnosis of infectious diseases is their ability to detect organisms that are difficult to culture or in patients who may have been pre-treated with empiric antibiotics. Most of the joint specimens included in our study were negative for any organism by traditional culture (60%), including all cases of *K. kingae* infections, which were only detected by the LDT-PCR.

In contrast to previous studies, our cohort included a significant number of osteoarticular specimens positive for *K. kingae* and *N. gonorrhoeae*, which are known to be difficult to isolate in culture. The JI Panel detected 15 out of 16 joint specimens positive for *K. kingae* (93.8%) and 3 out of 4 specimens positive for *N. gonorrhoeae* (75%), emphasizing the potential utility of this panel for identification of these fastidious pathogens. *K. kingae* was the most common pathogen detected in joint specimens in our cohort, consistent with recent studies reporting that over 50% of septic arthritis cases in children are caused by *K. kingae* (15). The inclusion of this target in the JI Panel offers a significant advantage in the rapid diagnosis of pediatric septic arthritis, especially since other FDA-cleared molecular assays for detection of *K. kingae* in synovial fluids are not available. This could be particularly advantageous for laboratories that lack the capability to test for *K. kingae* in-house using laboratory-developed molecular tests.

Multiple studies have reported lower sensitivities (41.7%–69%) (16–18) of the JI Panel when accounting for detection of off-panel organisms by culture, partly attributed to the inability of the JI Panel to detect significant pathogens associated with chronic prosthetic JIs such as *S. epidermidis* and *Cutibacterium acnes*. However, this limitation of the JI Panel is not as significant in pediatric patients who primarily present with acute osteoarticular infections of native joints caused primarily by on-panel target organisms. In our study, most of the off-panel organisms isolated in osteoarticular specimens likely represent skin or environmental culture contaminants, however we did not perform extensive chart review to confirm this.

Only two false positive results for *E. coli* and *Peptoniphilus* were observed in one hip synovial fluid specimen. *S. agalactiae*, *S. epidermidis* and *Prevotella bivia,* were recovered from this specimen by culture. The polymicrobial nature of this specimen may have impacted the specificity of the JI Panel in this case, a limitation that has been previously described with multiplex molecular assays (19, 20).

For pleural specimens, the JI Panel demonstrated an overall PPA of 93.8% and NPA of 99.6% for detection of on-panel organisms. Like osteoarticular specimens, culture positivity for pleural specimens in our cohort was low (22.8%). *S. pneumoniae* was the most common pathogen detected by the JI Panel with a PPA of 95.2% compared to SOC testing. Consistent with previous findings, the majority of *S. pneumoniae* cases were exclusively detected by the LDT-PCR (6).

There were several false positive JI Panel results identified in the testing of pleural specimens. This could be due to the detection of organisms that may be normal respiratory flora, but that did not grow in culture, including *H. influenzae*, *S. pyogenes*, *P. micra*, and *Streptococcus* spp. Interpretation of these molecular detections is challenging since some of these organisms (*H. influenzae* and *S. pyogenes*) are also important respiratory pathogens in children (21). Interestingly, higher detection rates of *H. influenzae, S. aureus,* and *Moraxella catarrhalis* in bronchoalveolar lavage specimens by a multiplex-PCR molecular panel compared to culture was not found to be associated with pneumonia in adults (22).

Despite the false positives observed in pleural specimens, the JI panel holds promise for influencing timely treatment decisions, particularly in culture negative cases. Its ability to accurately detect *S. pneumoniae,* the leading cause of complicated, community-acquired pneumonia in children, makes it particularly valuable. Indeed, utilization of a molecular assay for detection of *S. pneumoniae* in pleural fluids was shown to increase the likelihood of receiving optimal therapy and decrease the time to administration of appropriate antibiotics in a recent pediatric study (7).

The JI Panel has the added benefit of detecting some antimicrobial resistance determinants and has the potential to impact rapid escalation or de-escalation of antimicrobial therapy, as has been previously described for other rapid molecular panels (23). Although we were able to evaluate the performance of the *mecA* gene target for detection of methicillin resistance in *S. aureus* and the presence of CTX-M in one *E. coli* isolate*,* our evaluation of other antimicrobial resistance genes was limited, and further evaluation of these targets is warranted.

Limitations of this study include its retrospective nature, resulting in inherent biases due to the nonrandom selection of samples, and the off-label utilization of the JI Panel in osteoarticular abscesses and pleural specimens, including two specimens collected using ESwab transport media. Even though utilization of this panel with previously frozen and off-label specimens may have affected the performance of the assay, we believe that the analysis presented here is important to understand the strengths and weaknesses of the assay during its real-world application, as off-label specimens included in this study are routinely tested with the LDT-PCR.

In conclusion, the BioFire JI Panel has the potential to offer clinical benefits for the diagnosis of native septic arthritis and pleural space infections in children by enabling quicker clinical management decisions. Nonetheless, due to the inherent limitations of molecular panels which include a limited number of targets, adjunctive use with standard cultures and susceptibility testing is warranted. The hypothetical use of the JI Panel alongside culture for the specimens included in this study, would have resulted in a pathogen identification in 91.1% (51/56) of osteoarticular and 94.1% (32/34) of pleural specimens that were positive by the SOC composite results.

## SUPPLEMENTAL MATERIAL

Supplemental material jcm.00621-25-s0001.docx Organisms and antimicrobial resistance genes detected by the BIOFIRE JI Panel.

Table S2 jcm.00621-25-s0002.docx Diagnostic testing results for osteoarticular specimens.

Table S3 jcm.00621-25-s0003.docx Diagnostic testing results for pleural specimens.

ASM does not own the copyrights to Supplemental Material that may be linked to, or accessed through, an article. The authors have granted ASM a non-exclusive, world-wide license to publish the Supplemental Material files. Please contact the corresponding author directly for reuse.
