# Supplementary material for: Clinical performance of a syndromic panel for direct identification of pathogens and antimicrobial resistance markers in pediatric osteoarticular and pleural space infections
Source: J Clin Microbiol. 2025 Sep 2;63(11):e00621-25. doi: 10.1128/jcm.00621-25 (PMC12607902; doi:10.1128/jcm.00621-25)
Supplement: Supplemental material — Organisms and antimicrobial resistance genes detected by the BIOFIRE JI Panel. [file jcm.00621-25-s0001.docx]

| TABLE S1. Organisms and antimicrobial resistance genes detected by the BIOFIRE JI Panel | | | |
| --- | --- | --- | --- |
| **Gram-positive bacteria** | | | |
| *Anaerococcus prevotii/vaginalis* | *Parvimonas micra* | *Streptococcus* spp. | |
| *Clostridium perfringens* | *Peptoniphilus* spp. | *Streptococcus agalactiae* | |
| *Cutibacterium avidum/granulosum* | *Peptostreptococcus anaerobius* | *Streptococcus pneumoniae* | |
| *Enterococcus faecalis* | *Staphylococcus aureus* | *Streptococcus pyogenes* | |
| *Enterococcus faecium* | *Staphylococcus lugdunensis* |  | |
| *Finegoldia magna* |  |  | |
| **Gram-negative bacteria** | | | |
| *Bacteroides fragilis* | *Kingella kingae* | *Proteus* spp. | |
| *Citrobacter* spp. | *Klebsiella aerogenes* | *Pseudomonas aeruginosa* | |
| *Enterobacter cloacae* complex | *Klebsiella pneumoniae group* | *Salmonella* spp. | |
| *Escherichia coli* | *Morganella morganii* | *Serratia marcescens* | |
| *Haemophilus influenzae* | *Neisseria gonorrhoeae* |  | |
| **Yeast** | | | |
| *Candida* spp. |  |  |  |
| *Candida albicans* |  |  |  |
| **Antimicrobial resistance genes** | | | |
| CTX-M | *mecA/C* and MREJ (MRSA) | *vanA/B* | |
| IMP | NDM | VIM | |
| KPC | OXA-48-like |  |  |
