# Supplementary material for: Clinical performance of a syndromic panel for direct identification of pathogens and antimicrobial resistance markers in pediatric osteoarticular and pleural space infections
Source: J Clin Microbiol. 2025 Sep 2;63(11):e00621-25. doi: 10.1128/jcm.00621-25 (PMC12607902; doi:10.1128/jcm.00621-25)
Supplement: Table S2 — Diagnostic testing results for osteoarticular specimens. [file jcm.00621-25-s0002.docx]

| **Table S2.** Diagnostic testing results for osteoarticular specimens | | | | | | |
| --- | --- | --- | --- | --- | --- | --- |
| **#** | **Specimen Type** | **LDT-PCR** | **Culture^a^** | **Ancillary testing^b^** | **SOC composite** | **BioFire JIP Results** |
| J1 | Synovial Fluid, Knee, L | MSSA | MSSA | N/A | MSSA | MSSA |
| J2 | Synovial Fluid, Shoulder, R | MSSA | MSSA | N/A | MSSA | MSSA |
| J3 | Synovial Fluid, Elbow, R | MRSA | Negative | N/A | MRSA | MRSA |
| J4 | Synovial Fluid, Knee, R | MRSA | MRSA | N/A | MRSA | MRSA |
| J5 | Synovial Fluid, Knee, R | MRSA | MRSA | N/A | MRSA | MRSA |
| J6 | Synovial Fluid, Ankle, R | *K. kingae* | Negative | N/A | *K. kingae* | *K. kingae* |
| J7 | Synovial Fluid, Knee, L | *K. kingae* | Negative | N/A | *K. kingae* | *K. kingae* |
| J8 | Synovial Fluid, Knee, L | *K. kingae* | *Bacillus* spp. | N/A | *K. kingae*  *Bacillus* spp. | *K. kingae* |
| J9 | Abscess, Knee, R | *K. kingae* | Negative | N/A | *K. kingae* | *K. kingae* |
| J10 | Synovial Fluid, Knee, R | *K. kingae* | Negative | N/A | *K. kingae* | Negative |
| J11 | Synovial Fluid, Knee, L | *K. kingae* | Negative | N/A | *K. kingae* | *K. kingae* |
| J12 | Synovial Fluid, Knee, R | *K. kingae* | Negative | N/A | *K. kingae* | *K. kingae* |
| J13 | Synovial Fluid, Elbow, R | *S. pneumoniae* | *S. pneumoniae* | N/A | *S. pneumoniae* | *S. pneumoniae* |
| J14 | Synovial Fluid, Knee, L | *S. pyogenes* | Negative | N/A | *S. pyogenes* | Negative |
| J15 | Synovial Fluid, Ankle, L | *S. pyogenes* | *S. pyogenes* | N/A | *S. pyogenes* | *S. pyogenes* |
| J16 | Synovial Fluid, Elbow, L | Negative | Negative | *N. gonorrhoeae* | *N. gonorrhoeae* | *N. gonorrhoeae* |
| J17 | Synovial Fluid, Knee, R | Negative | *S. anginosus* | N/A | *S. anginosus* | Negative |
| J18 | Synovial Fluid, Shoulder, R | Negative | *S. agalactiae* | N/A | *S. agalactiae* | *S. agalactiae* |
| J19 | Synovial Fluid, Hip, L | Negative | ESBL-producing  *E. coli* | N/A | ESBL-producing  *E. coli* | *E. coli*  CTX-M |
| J20 | Synovial Fluid, Knee, L | Negative | *S. marcescens* | N/A | *S. marcescens* | *S. marcescens* |
| J21 | Synovial Fluid, Shoulder, R | Negative | *E. coli* | N/A | *E. coli* | *E. coli* |
| J22 | Synovial Fluid, Hip, L | Negative | *Bacillus* spp. | N/A | *Bacillus* spp. | Negative |
| J23 | Synovial Fluid, Knee, L | *K. kingae* | *Acinetobacter* spp. | N/A | *K. kingae*  *Acinetobacter* spp. | *K. kingae* |
| J24 | Synovial Fluid, Knee, R | Negative | Negative | *N. gonorrhoeae* | *N. gonorrhoeae* | Negative |
| J25 | Synovial Fluid, Knee, L | Negative | Negative | N/A | Negative | Negative |
| J26 | Synovial Fluid, Knee, L | Negative | Negative | N/A | Negative | Negative |
| J27 | Synovial Fluid, Ankle, R | Negative | Negative | N/A | Negative | Negative |
| J28 | Synovial Fluid, Knee, R | Negative | Negative | N/A | Negative | Negative |
| J29 | Synovial Fluid, Knee, L | Negative | N/A | N/A | Negative | Negative |
| J30 | Synovial Fluid, Hip, R | Negative | Negative | N/A | Negative | Negative |
| J31 | Synovial Fluid, Hip, L | Negative | Negative | N/A | Negative | Negative |
| J32 | Synovial Fluid, Knee, R | Negative | Negative | N/A | Negative | Negative |
| J33 | Synovial Fluid, Ankle, R | Negative | Negative | N/A | Negative | Negative |
| J34 | Synovial Fluid, Shoulder, R | Negative | Negative | N/A | Negative | Negative |
| J35 | Synovial Fluid, Hip, R | Negative | Negative | N/A | Negative | Negative |
| J36 | Synovial Fluid, Synovium | Negative | Negative | *N. gonorrhoeae* | *N. gonorrhoeae* | *N. gonorrhoeae* |
| J37 | Synovial Fluid, Knee, R | Negative | Negative | *N. gonorrhoeae* | *N. gonorrhoeae* | *N. gonorrhoeae* |
| J38 | Synovial Fluid, Knee, L | Negative | Negative | N/A | Negative | Negative |
| J39 | Abscess, Ankle, R | *K. kingae* | Negative | *K. kingae* | *K. kingae* | *K. kingae* |
| J40 | Synovial Fluid, Hip, R | Negative | *Salmonella* spp. | Negative | *Salmonella* spp. | *Salmonella* spp. |
| J41 | Synovial Fluid, Leg, L | MSSA | Negative | N/A | MSSA | Negative |
| J42 | Synovial Fluid, Knee, L | Negative | Negative | N/A | Negative | Negative |
| J43 | Synovial Fluid, Wrist, L | *S. pyogenes* | Negative | N/A | *S. pyogenes* | *S. pyogenes* |
| J44 | Synovial Fluid, Ankle, R | Negative | Negative | N/A | Negative | Negative |
| J45 | Synovial Fluid, Hip, L | Negative | *S. auricularis*  *M. luteus* | N/A | *S. auricularis*  *M. luteus* | Negative |
| J46 | Synovial Fluid, Knee, R | MSSA | MSSA | N/A | MSSA | MSSA |
| J47 | Synovial Fluid, Hip, R | Negative | *S. agalactiae*  *S. epidermidis*  *Prevotella bivia* | N/A | *S. agalactiae,*  *S. epidermidis,*  *Prevotella bivia* | *S. agalactiae*  *Peptoniphilus*  *E. coli* |
| J48 | Synovial Fluid, Elbow, R | Negative | *P. aeruginosa* | N/A | *P. aeruginosa* | *P. aeruginosa* |
| J49 | Synovial Fluid, Ankle, L | *K. kingae* | Negative | N/A | *K. kingae* | *K. kingae* |
| J50 | Synovial Fluid, Leg, L | *S. pyogenes* | Negative | N/A | *S. pyogenes* | *S. pyogenes* |
| J51 | Synovial Fluid, Hip, L | *S. pyogenes* | S. pyogenes | N/A | *S. pyogenes* | *S. pyogenes* |
| J52 | Synovial Fluid, Knee, R | *K. kingae* | Negative | N/A | *K. kingae* | *K. kingae* |
| J53 | Abscess, Hip, L | MSSA | MSSA | N/A | MSSA | MSSA |
| J54 | Synovial Fluid | *K. kingae* | Negative | N/A | *K. kingae* | *K. kingae* |
| J55 | Synovial Fluid, Ankle, L | MSSA | MSSA | N/A | MSSA | MSSA |
| J56 | Synovial Fluid, Knee, L | Negative | Negative | N/A | Negative | Negative |
| J57 | Synovial Fluid, Synovium  (E-Swab) | MSSA | Negative | N/A | MSSA | Negative |
| J58 | Synovial Fluid, Knee, L | Negative | Negative | N/A | Negative | Negative |
| J59 | Synovial Fluid, Knee, L | Negative | Negative | N/A | Negative | Negative |
| J60 | Synovial Fluid, Knee, L | *S. pneumoniae* | Negative | *S. pneumoniae* | *S. pneumoniae* | *S. pneumoniae* |
| J61 | Synovial fluid (E-Swab) | Negative | Not available | N/A | Negative | Negative |
| J62 | Synovial Fluid, Knee, L | *S. pyogenes* | Negative | N/A | *S. pyogenes* | *S. pyogenes* |
| J63 | Synovial Fluid, Knee, R | Negative | Negative | N/A | Negative | Negative |
| J64 | Synovial Fluid, Knee, L | *S. pyogenes* | *S. pyogenes* | N/A | *S. pyogenes* | *S. pyogenes* |
| J65 | Abscess, Humerus, R | MSSA | MSSA | N/A | MSSA | MSSA |
| J66 | Synovial Fluid, Elbow, R | MSSA | MSSA | N/A | MSSA | MSSA |
| J67 | Abscess, Radius, R | *S. pyogenes* | *S. pyogenes* | N/A | *S. pyogenes* | *S. pyogenes* |
| J68 | Synovial Fluid, Hip, L | MSSA | MSSA | N/A | MSSA | MSSA |
| J69 | Synovial Fluid, Knee, L | *K. kingae* | Negative | N/A | *K. kingae* | *K. kingae* |
| J70 | Synovial Fluid, Knee, R | *S. pyogenes* | Negative | N/A | *S. pyogenes* | *S. pyogenes* |
| J71 | Abscess, Foot, L | *K. kingae* | Negative | N/A | *K. kingae* | *K. kingae* |
| J72 | Synovial Fluid, Elbow, R | Negative | *Salmonella* spp. | N/A | *Salmonella* spp. | *Salmonella* spp. |
| J73 | Abscess, Elbow, L | *S. pyogenes* | *S. pyogenes* | N/A | *S. pyogenes* | *S. pyogenes* |
| J74 | Synovial Fluid, Knee, R | *K. kingae* | Negative | N/A | *K. kingae* | *K. kingae* |
| J75 | Synovial Fluid, Hip, L | *K. kingae* | Negative | *K. kingae* | *K. kingae* | *K. kingae* |
| J76 | Abscess, Knee, R | MRSA | MRSA | N/A | MRSA | MSSA |
| J77 | Synovial Fluid, Knee, L | *S. pyogenes* | Negative | N/A | *S. pyogenes* | *S. pyogenes* |
| ^a^Anaerobic culture results are included if available.  ^b^Ancillary testing includes positive blood cultures, 16s rRNA PCR and sequencing (University of Washington) and *Kingella kingae*-specific PCR (Quest laboratories) performed in joint specimens.  N/A: not applicable; LDT: lab-developed test; MSSA: methicillin-susceptible *S. aureus*; MRSA: methicillin-resistant *S. aureus* | | | | | | |
