# Supplementary material for: Clinical performance of a syndromic panel for direct identification of pathogens and antimicrobial resistance markers in pediatric osteoarticular and pleural space infections
Source: J Clin Microbiol. 2025 Sep 2;63(11):e00621-25. doi: 10.1128/jcm.00621-25 (PMC12607902; doi:10.1128/jcm.00621-25)
Supplement: Table S3 — Diagnostic testing results for pleural specimens. [file jcm.00621-25-s0003.docx]

| **Table S3.** Diagnostic testing results for pleural specimens | | | | | | |
| --- | --- | --- | --- | --- | --- | --- |
| **#** | **Specimen Type** | **LDT-PCR** | **Culture^a^** | **Ancillary testing^b^** | **SOC composite** | **BioFire JIP** |
| P1 | Body Fluid, Pleural, R | *S. pneumoniae* | Negative | N/A | *S. pneumoniae* | *S. pneumoniae* |
| P2 | Body Fluid, Pleural, R | Negative | *Prevotella oralis S. constellatus* | N/A | *Prevotella oralis*  *S. constellatus* | *Parvimonas micra Streptococcus* spp. |
| P3 | Body Fluid, Pleural, L | Negative | Negative | N/A | Negative | Negative |
| P4 | Body Fluid, Pleural, L | *S. pneumoniae* | *S. pneumoniae* | N/A | *S. pneumoniae* | *S. pneumoniae* |
| P5 | Body Fluid, Pleural, L | Negative | Negative | N/A | Negative | Negative |
| P6 | Body Fluid, Pleural, L | *S. pneumoniae* | Negative | N/A | *S. pneumoniae* | *S. pneumoniae* |
| P7 | Body Fluid, Pleural, L | *S. pneumoniae* | Negative | N/A | *S. pneumoniae* | *S. pneumoniae* |
| P8 | Body Fluid, Pleural, R | *S. pneumoniae* | Negative | N/A | *S. pneumoniae* | *S. pneumoniae* |
| P9 | Body Fluid, Pleural, R | Negative | Negative | N/A | Negative | Negative |
| P10 | Body Fluid, Pleural, L | *S. pneumoniae* | Negative | N/A | *S. pneumoniae* | *S. pneumoniae* |
| P11 | Body Fluid, Pleural, R | Negative | Negative | N/A | Negative | Negative |
| P12 | Body Fluid, Pleural, R | *S. pneumoniae* | Negative | N/A | *S. pneumoniae* | *S. pneumoniae* |
| P13 | Body Fluid, Pleural, R | *S. pneumoniae* | Negative | N/A | *S. pneumoniae* | *S. pneumoniae* |
| P14 | Body Fluid, Pleural, R | *S. pneumoniae* | Negative | N/A | *S. pneumoniae* | *S. pneumoniae* |
| P15 | Body Fluid, Pleural, R | *S. pneumoniae* | Negative | N/A | *S. pneumoniae* | *S. pneumoniae* |
| P16 | Body Fluid, Pleural, L | *S. pneumoniae* | *S. pneumoniae* | N/A | *S. pneumoniae* | *S. pneumoniae* |
| P17 | Body Fluid, Pleural, R | *S. pneumoniae* | Negative | N/A | *S. pneumoniae* | *S. pneumoniae* |
| P18 | Body Fluid, Pleural, R | Negative | Negative | N/A | Negative | Negative |
| P19 | Body Fluid, Pleural, R | *S. pneumoniae* | Negative | N/A | *S. pneumoniae* | *S. pneumoniae*  *H. influenzae* |
| P20 | Body Fluid, Pleural, R | Negative | Negative | N/A | Negative | Negative |
| P21 | Body Fluid, Pleural, R | *S. pneumoniae* | Negative | N/A | *S. pneumoniae* | *S. pneumoniae* |
| P22 | Body Fluid, Pleural, L | Negative | Negative | N/A | Negative | Negative |
| P23 | Body Fluid, Pleural, R | Negative | Negative | N/A | Negative | Negative |
| P24 | Body Fluid, Pleural, L | Negative | Negative | N/A | Negative | Negative |
| P25 | Body Fluid, Pleural, R | MSSA | MSSA | N/A | MSSA | MSSA |
| P26 | Body Fluid, Pleural, L | MSSA | Negative | N/A | MSSA | Negative |
| P27 | Body Fluid, Pleural, R | Negative | Negative | N/A | Negative | Negative |
| P28 | Body Fluid, Pleural, R | Negative | Negative | N/A | MSSA | Negative |
| P29 | Body Fluid, Pleural, R | Negative | Negative | N/A | Negative | Negative |
| P30 | Body Fluid, Pleural, L | Negative | Negative | N/A | Negative | Negative |
| P31 | Body Fluid, Pleural, L | Negative | Negative | N/A | Negative | Negative |
| P32 | Body Fluid, Pleural, L | *S. pyogenes* | Negative | N/A | *S. pyogenes* | *S. pyogenes* |
| P33 | Body Fluid, Pleural, R | MRSA | Negative | N/A | MRSA | Negative |
| P34 | Body Fluid, Pleural, R | Negative | Negative | N/A | Negative | *Streptococcus* spp.  *Parvimonas micra^c^* |
| P35 | Body Fluid, Pleural, L | Negative | Not available | N/A | Negative | Negative |
| P36 | Body Fluid, Pleural, R | Negative | Negative | N/A | Negative | Negative |
| P37 | Body Fluid, Pleural, R | Negative | Negative | N/A | Negative | Negative |
| P38 | Body Fluid, Pleural, L | Negative | Negative | N/A | Negative | Negative |
| P39 | Body Fluid, Pleural, L | *S. pyogenes* | Negative | N/A | *S. pyogenes* | *S. pyogenes* |
| P40 | Body Fluid, Pleural, L | Negative | Negative | N/A | Negative | Negative |
| P41 | Body Fluid, Pleural, L | *S. pneumoniae* | Negative | N/A | *S. pneumoniae* | *S. pneumoniae* |
| P42 | Body Fluid, Pleural, L | Negative | Not available | N/A | Negative | Negative |
| P43 | Body Fluid, Pleural, R | *S. pyogenes* | Negative | N/A | *S. pyogenes* | *S. pyogenes* |
| P44 | Body Fluid, Pleural, R | *S. pyogenes* | Negative | N/A | *S. pyogenes* | *S. pyogenes* |
| P45 | Body Fluid, Pleural, L | Negative | Negative | N/A | Negative | Negative |
| P46 | Body Fluid, Pleural, R | *S. pyogenes* | *S. pyogenes* | N/A | *S. pyogenes* | *S. pyogenes* |
| P47 | Body Fluid, Pleural, R | *S. pyogenes* | *S. pyogenes* | N/A | *S. pyogenes* | *S. pyogenes* |
| P48 | Body Fluid, Pleural, R | S. pneumoniae | H. influenzae | N/A | S. pneumoniae  H. influenzae | H. influenzae |
| P49 | Body Fluid, Pleural, R | S. pneumoniae | S. epidermidis | N/A | S. pneumoniae  S. epidermidis | S. pneumoniae |
| P50 | Abscess, Pleural, L | Negative | *Parvimonas micra*  *Prevotella* spp.  *S. constellatus*  *G. adiacens*  *A. odontolyticus,*  *S. anginosus* | N/A | *Parvimonas micra*  *Prevotella* spp.  *S. constellatus*  *G. adiacens*  *A. odontolyticus*  *S. anginosus* | *Parvimonas micra Streptococcus* spp.  *S. pyogenes*  *H. influenzae* |
| P51 | Body Fluid, Pleural, R | Negative | Negative | N/A | Negative | Negative |
| P52 | Body Fluid, Pleural, R | Negative | Negative | N/A | Negative | Negative |
| P53 | Body Fluid, Pleural, L | *S. pneumoniae* | *S. pneumoniae* | N/A | *S. pneumoniae* | *S. pneumoniae* |
| P54 | Body Fluid, Pleural, R | *S. pneumoniae* | *S. pneumoniae* | N/A | *S. pneumoniae* | *S. pneumoniae* |
| P55 | Body Fluid, Pleural, L | Negative | Negative | N/A | Negative | Negative |
| P56 | Body Fluid, Pleural, L | *S. pneumoniae* | Negative | N/A | *S. pneumoniae* | *S. pneumoniae* |
| P57 | Body Fluid, Pleural, L | *S. pneumoniae* | Negative | N/A | *S. pneumoniae* | *S. pneumoniae* |
| P58 | Body Fluid, Pleural, L | MRSA | MRSA | N/A | MRSA | MRSA |
| P59 | Body Fluid, Pleural, L | Negative | *H. influenzae* | *S. mitis* | *H. influenzae*  *S. mitis* | *H. influenzae*  *Streptococcus* spp. |
| ^a^Anaerobic culture results are included if available.  ^b^Ancillary testing includes 16s rRNA PCR and sequencing (University of Washington).  ^c^ This *Parvimonas micra* result by the JI Panel was excluded from the performance analysis because an anaerobic culture result was not available for this specimen.  N/A: not applicable; LDT: lab-developed test; MSSA: methicillin-susceptible *S. aureus*; MRSA: methicillin-resistant *S. aureus* | | | | | | |
